# Nanoparticle manipulation by thermal gradient

**DOI:** 10.1186/1556-276X-7-154

**Published:** 2012-02-26

**Authors:** Ning Wei, Hui-Qiong Wang, Jin-Cheng Zheng

**Affiliations:** 1 Department of Physics and Institute of Theoretical Physics and Astrophysics, Xiamen University, Xiamen, 361005, China; 2 Fujian Key Lab of Semiconductor Materials and Applications, Xiamen University, Xiamen, 361005, China; 3 Fujian Provincial Key Laboratory of Theoretical and Computational Chemistry, Xiamen University, Xiamen, 361005, China

**Keywords:** molecular dynamics simulation, thermal gradient, thermophoresis, manipulation, carbon nanotube, mass transport

## Abstract

A method was proposed to manipulate nanoparticles through a thermal gradient. The motion of a fullerene molecule enclosed inside a (10, 10) carbon nanotube with a thermal gradient was studied by molecular dynamics simulations. We created a one-dimensional potential valley by imposing a symmetrical thermal gradient inside the nanotube. When the temperature gradient was large enough, the fullerene sank into the valley and became trapped. The escaping velocities of the fullerene were evaluated based on the relationship between thermal gradient and thermophoretic force. We then introduced a new way to manipulate the position of nanoparticles by translating the position of thermostats with desirable thermal gradients. Compared to nanomanipulation using a scanning tunneling microscope or an atomic force microscope, our method for nanomanipulation has a great advantage by not requiring a direct contact between the probe and the object.

## Background

Manipulation of atoms using a scanning tunneling microscope (STM) or an atomic force microscope (AFM) reveals the new era of nanotechnology or nanodesign [1]. Atoms can now be arranged on demand. However, these approaches require a direct contact between the probe of STM/AFM and the object, which is of strong instrumental dependence and thus greatly restrains the manipulation. It is therefore desirable to seek new methods to manipulate nanoparticles without contact. In this work, we demonstrated that the mechanism of thermophoresis, in which case, atoms move opposite to the thermal gradient, could be utilized for nanoparticle manipulation.

Thermophoresis was actually discovered in the nineteenth century [2] which was induced by thermodiffusion. It was originally used to provide driving forces for molecules to move in fluids or gases [3,4]. Recently, thermophoresis has been used for the manipulation and stretching of DNA [4,5]. Carbon nanotubes (CNTs) have been selected as ideal 'transfer belts' for transferring gases and liquids by thermophoresis due to their atomically smooth surfaces and solid walls [6-11]. It is not until very recently that thermophoresis is found applicable on large molecular clusters or nanostructures. Experimentally, driven by thermal gradients, short CNTs are found to move relative to coaxial, longer CNTs [12,13]; they can act as shuttles or capped capsules inside the longer CNTs [13] or as cargos attached to the outer walls of the longer CNTs [12]. These short CNTs move while confined and guided by the host CNTs, and the motions could be translational or helical depending on the chirality of the CNTs [12]. These exciting experimental results have stimulated much theoretical research on thermophoretic transport. From the theoretical calculations, all nanoparticles enclosed in the CNTs, including short CNTs [14], fullerene [15], gold nanoparticles [16,17], and water droplets [10,11] are found to move opposite to the imposed thermal gradient, and the terminal velocities are linearly proportional to the gradient. In addition, Zambrano et al. [14] discovered that the magnitude of thermophoretic force was not only related to the temperature gradient, but also dependent on the velocities of the transported molecules. At the same temperature gradient, the faster the motion, the smaller the thermophoretic force. Furthermore, thermophoresis is becoming one of the main approaches for cargo transport at nanoscale [18]. Nevertheless, in all these previous reports, thermophoretic force is basically used to provide driving forces for the transportation of molecular linear motors.

In this paper, we extend the study of thermophoresis on nanotechnology in the aspect that not only the motions, but also the physical positions of the nanoparticles can be manipulated. Based on the mechanism of thermophoresis, we propose a model in which a fullerene is placed inside a CNT and subjected to a eudipleural thermal gradient. The cold region is in the middle, and the hot regions are in both ends, with periodic conditions along the axial direction. The fullerene inside will then be pushed to the middle cold region by the thermophoretic forces. In this case, we create a potential valley based on the temperature gradient. When the potential barrier is high enough, the fullerene will be trapped inside the potential valley. More interestingly, the trapped fullerene can also move as the cold source is shifted. Our results indicate the feasibilities of manipulating nanoparticles utilizing thermophoretic forces. The application of thermophoresis can then be extended from the thermal driving of molecular linear motors to thermal restrain using potential valleys and to thermal nanomanipulation.

## Methods

This C_60_-CNT system was modeled with adaptive intermolecular reactive empirical bond order (AIREBO) potential [19], which deals with covalent carbon-carbon bonding interactions determined by the well-established REBO potential, while the nonbonded interaction between the carbon atoms of fullerene and those of CNT was described by the Lennard-Jones potential, as implemented in the LAMMPS package [20], which can be used to well describe the thermal properties of CNT [21], graphene [22], or graphene-nanotube 3D networks [23] or the mechanical properties of knitted graphene [24], with a time step 0.5 fs. Before the nonequilibrium simulation, the system temperature is set to 400 K by a Nosé-Hoover thermostat [25,26] for 500 ps with a coupling constant of 0.1 ps. After the system reaches equilibrium, heat flux is imposed by the thermostats, where the cold region is selected in the middle and the hot regions are in both ends of CNT. The heat flux is injected/subtracted from carbon atoms of the hot/cold regions of CNT [27], which are realized by rescaling velocities of carbon atoms according to:

(1) $$v_{i}^{\prime }=v_{T}+\alpha \left(v_{i}-v_{T}\right),$$

where $v_{i}^{\prime }$ and *v_i _*are the new and old velocities of thermostat atoms, respectively, *v_T _*is the velocity of the center of mass of the thermostat, and *α *is the rescaling factor:

(2) $$\alpha =\sqrt{1\pm \frac{\Delta \varepsilon }{E_{\text{R}}}},$$

where ± Δ*ε *is the energy injected/subtracted from specified atoms. *E*_R _is the relative kinetic energy, which is defined as:

(3) $$E_{\text{R}}=\underset{i}{\sum }\frac{1}{2}m_{i}\left(v_{i}^{2}-v_{T}^{2}\right).$$

In our simulation, the heat flux is fixed as:

(4) $$J=\frac{\Delta \varepsilon }{2A\Delta t},$$

and the value is controlled by Δ*ε*. In order to calculate the temperature distribution in the outer CNT, the CNT is divided into 50 slabs along the axial direction. The temperature of each slab is computed by the following:

(5) $$T_{i}\left(\text{slab}\right)=\frac{2}{3Nk_{\text{B}}}\underset{i}{\sum }\frac{p_{j}^{2}}{2m},$$

where *T_i_*(slab) is the temperature of the *i*th slab, *N *is the number of carbon atoms in this slab, *k*_B _is the Boltzmann constant, and *p_j _*is the momentum of atom *j*. The nonequilibrium molecular dynamics (NEMD) simulation is performed under NEV ensemble for 5 ns.

## Results and discussions

**Figure 1:**
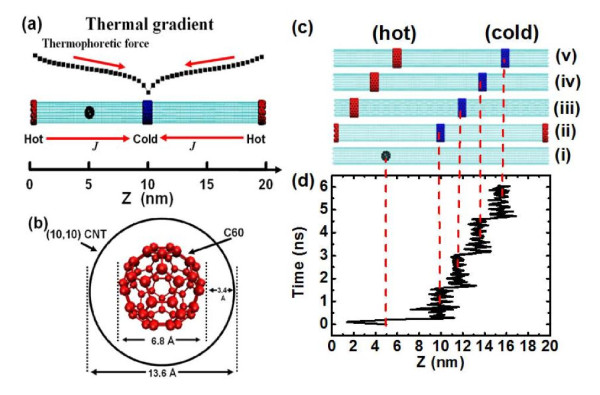
**Illustration of fullerene nanomanipulation inside CNT through the translation of cold and hot thermal sources**. (**a**) Illustration of the simulation model. A fullerene is enclosed in a (10, 10) CNT which is 20.0 nm in length and subjected into a symmetrical thermal gradient, with the hot region at both ends and the cold region at the middle of CNT. Fullerene is placed at 5.0 nm away from the tube ends. (**b**) View of the cross section model. The closest distance between the carbon atoms of fullerene to the CNT wall is 3.4 Å, similar to the interlayer distance of graphite. (**c**, **d**) Nanomanipulation is achieved through the translation of cold and hot thermal sources, correspondingly. The initial position of C_60 _was placed at one-fourth of the nanotube, middle of cold and hot sources. The translation of thermal sources was performed three times, each to move about 1.2 nm along the nanotube, while keeping a fixed distance between cold and hot sources. The C_60 _molecular is trapped around the cold source position and moves with the thermostats.

In the molecular dynamics simulations, we adapted the peapod model [28,29] for a fullerene (C_60_) encapsulated into a (10, 10) CNT with a length of approximately 20 nm. As shown in Figure 1, the diameters of the C_60 _molecule and the (10, 10) CNT are 6.8 and 13.6 Å, respectively. As a result, the closest distance between the carbon atoms of the fullerene to the CNT wall is 3.4 Å, similar to the interlayer distance of graphite, and is expected to be the most effective distance for C_60 _to be encapsulated inside the CNT [15,30]. Heat flux was subjected to the CNT through the traditional momentum rescaling method [27,31], injected/subtracted energy of 7.0 eV/ps from carbon atoms of hot/cold thermostats of CNT, after the system reaches equilibrium at 400 K. The initial cold thermostat is in the middle, and the hot thermostats are in both ends, with periodic conditions along the axial direction as shown in Figure 1a. After 1.5 ns, the thermostats, both the cold and hot ones, translate 1.2 nm along the nanotube axially toward a right direction, correspondingly, while maintaining a fixed distance between cold and hot thermostats. Such a translation is conducted three times to move forward (see Figure 1c). The trajectories of the center of mass (COM) of the C_60 _as a function of simulation time are shown in Figure 1d. It is interesting to find that in the NEMD simulation, C_60 _is confined in the cold region and oscillates within a range of △*z *about ± 1.0 nm along the axial direction. When the thermostats shift along the tube axially, C_60 _moves together with the cold one. The reason is that in the nonequilibrium system, the encapsulated C_60 _cluster suffers the thermophoretic force in the direction opposite to that of the thermal gradient, pointing from the hot region (ends) to the cold region (middle). Hence, a one-dimensional potential valley is created inside the CNT by the thermal gradient. When the kinetic energy of the fullerene is less than the magnitude of potential barrier energy, C_60 _will be trapped and its motion will be confined inside the valley (the cold region). In our symmetrical thermal gradient simulations, the 'potential valley' is created by the thermophoretic force. Therefore, we manipulate the nanoparticle by utilizing the idea of potential valley by translating the corresponding thermal source locations.

**Figure 2:**
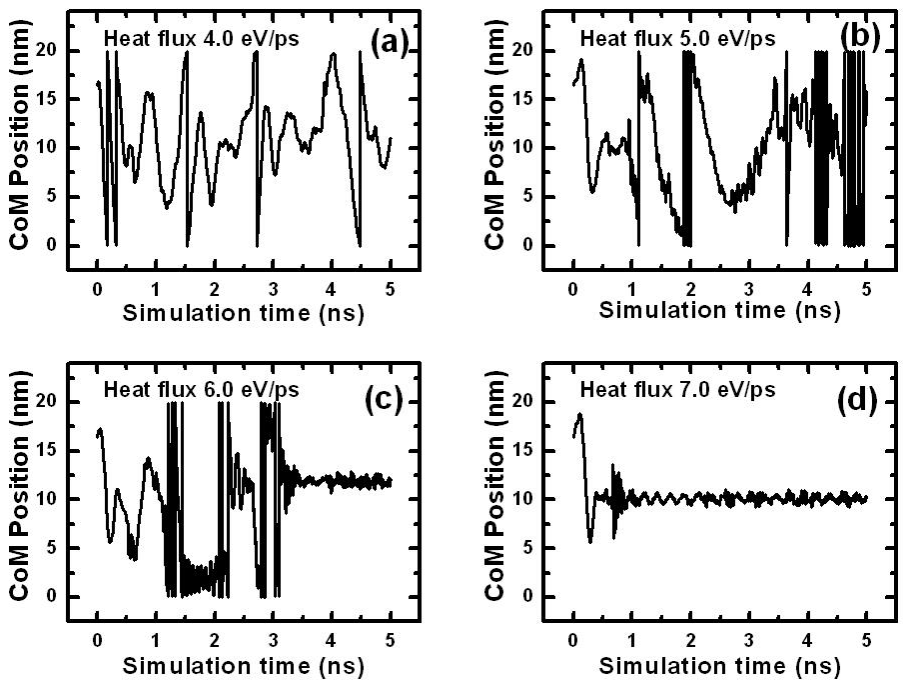
**The positions of COM of C_60 _as a function of simulation time**. Under heat flux equal to (**a**) 4.0, (**b**) 5.0, (**c**) 6.0, and (**d**) 7.0 eV/ps. The length scale of COM is in nanometers, and the time scale is in nanoseconds.

In order to further examine our idea of the artificial potential valley by thermal gradient, several magnitudes of heat fluxes, i.e., 4.0, 5.0, 6.0, and 7.0 eV/ps are considered, with the initial locations of thermostats marked at the background temperature of 400 K. The COMs of the C_60 _as a function of simulation time are shown in Figure 2. The C_60 _cluster is bounced inside the CNT randomly, rather than doing a perfect harmonic oscillation. The main reason is that the driving force, thermophoretic force, is not uniform but is associated with the temperature gradient. We can see that when subjected to heat fluxes of 6.0 and 7.0 eV/ps, C_60 _is confined in a △*z *range of around 1 nm within our simulation time of 5 ns. It is noticed that the capture time, from the beginning of the simulation to the moment when C_60 _is confined by the thermal gradient, depends on the magnitude of heat flux. The greater the subjected heat flux, the sooner C_60 _will be trapped, e.g., the time is 3.5 ns for the subjected heat flux of 6.0 eV/ps and 0.8 ns for 7.0 eV/ps. On the other hand, when the fullerene's kinetic energy is larger than the potential barrier energy, the molecule can escape the valley and move along the whole CNT, as shown in Figure 2. This is due to the fact that the thermophoretic force increases with the thermal gradient, which also increases with heat flux. It is worth pointing out that after the C_60 _is trapped, the interaction between C_60 _and CNT will be enhanced and thus will influence the temperature distribution in CNT. The temperature of the cold region will become even lower, resulting in a greater thermal gradient.

**Figure 3:**
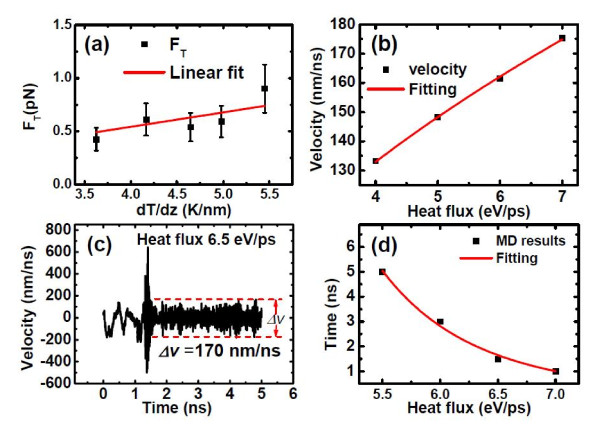
**The thermophoretic force, escaping velocity, and capture time of C_60 _inside CNT**. (**a**) Thermophoretic force as a function of the temperature gradient. The average system temperature is 400 K. An approximate linear relationship of the magnitude of the thermophoretic force and the thermal gradient was observed as $F_{\text{T}}=k_{\text{F}}\frac{dT}{dz}$. (**b**) The escaping velocity of C_60 _was evaluated by calculating the required energy of C_60 _from the cold bottom to the hot top according to the relationship of thermophoretic force and thermal gradient, which were clearly described in Additional file 1. (**c**) Velocity of C_60 _under the heat flux of 6.5 eV/ps. (**d**) Capture time of C_60 _as a function of heat flux.

The thermophoretic force is calculated by integrating the interaction forces between the outer nanotube and each atom of C_60_. Since the thermophoretic force varies with the thermal gradient, which is thermal gradient-dependent, when calculating the thermophoretic force for a certain thermal gradient, the motion of C_60 _is restrained along the nanotube's axial direction. This is done by rescaling the momentum of atoms of C_60_, so their velocities along the axial direction are zero. We record the interaction force between the nanotube wall and C_60 _within 1.0 ps upon the imposition of the thermal gradient for each simulation. The interaction force between the outer nanotube and each atom of C_60 _has an anti-symmetry distribution, and the net result is the thermophoretic force. An averaged value is obtained from five independent simulations. However, the thermophoretic force obtained by this approach is greater than that in the real case when the C_60 _molecule is moving instead of being restrained. This is because the thermophoretic force is not only related to the thermal gradient, but also velocity-dependent. A larger velocity of C_60 _induces a smaller thermophoretic force [14]. The error bar of the thermophoretic force comes from the systemic thermal fluctuation. An approximate linear relationship of the magnitude of the thermophoretic force (*F*_T_, in piconewton) and thermal gradient ($\frac{dT}{dz}$, in kelvin per nanometer) was fitted to be

(6) $$F_{\text{T}}=k_{\text{F}}\frac{dT}{dz},$$

where *k*_F _is 0.1356 (Figure 3a), which is consistent with those reported previously [10,11,32]. The magnitude of the thermophoretic force of C_60 _is of the same order as in some literatures [10,11], while smaller than the values presented by Hou et al. [32]. Such discrepancy could be due to the different lengths of host CNTs and the size of encapsulated cluster used in the calculations [32] compared with those of ours.

As seen from the above simulations, when the induced heat flux is large enough, e.g., larger than 6.0 eV/ps, C_60 _can be trapped by the thermal potential barrier in less than 5.0 ns. One might then wonder: at what velocity can C_60 _escape from the one-dimensional potential valley after being trapped by the thermal gradient? Here, we define the escaping velocity as the minimum velocity required for C_60 _to escape from the bottom of the potential valley (cold region) to the top of the valley (hot region). The energy of the one-dimensional potential valley is calculated according to the thermal gradient profile and the relationship between thermal gradient and thermophoretic force, which are well described in Additional file 1. The escaping velocity *v*_escape _is thus obtained from the kinetic equation:

(7) $$E_{\text{V}}=\frac{1}{2}m_{{\text{C}}_{60}}v_{\text{escape}}^{2},$$

where *E*_v _is the potential valley's energy, and $m_{{\text{C}}_{60}}$ is the mass of C_60_. A relationship between the escaping velocity *v*_escape _(in nanometers per nanosecond) and the applied heat flux *J *(in electron volts per picosecond) is also fitted to be:

(8) $$v_{\text{escape}}^{}=k_{v}\cdot J^{b_{v}},$$

where *k_v _*is 67.35 and *b_v _*is 0.49 (Figure 3b). In order to further verify this power-law relationship, we conduct a new simulation with the CNT subjected to a heat flux of 6.5 eV/ps, and the velocity of C_60 _as a function of simulation time is shown in Figure 3c: C_60 _was trapped by the thermal gradient potential valley in 1.5 ns, and the velocity of C_60 _after being trapped was about 170 nm/ns, well consistent with what is shown in Figure 3b. The relationship between the capture time *T*_trap _(in nanoseconds) of C_60 _and the magnitude of imposed heat flux *J *(in electron volts per picosecond) can be fitted into a power-law curve as:

(9) $$T_{trap}=k_{T}\cdot J^{b_{T}},$$

where *k_T _*is 449,384.5 and *b_T _*is -6.68 (Figure 3d). It would have been desirable to find out the relationship between the capture time of C_60 _and the thermal gradient, but this is difficult to obtain because of the position dependence of the thermal gradient. Note that the thermal gradients created in our NEMD simulations are higher than those used in the experiments [12,13], where the thermal gradient was created by Joule heating [12] and/or the electron beam of a transmission electron microscope [13], which was roughly in the range of 1 to 3 K/nm. In our NEMD simulations, it would be time-consuming to trap the molecule in such a low thermal gradient. Therefore, our simple power-law relationship as described above can be used to provide some theoretical predictions for experimental exploitation, e.g., with a low thermal gradient of 1 to 3 K/nm as used in the previous experiments [12,13]; the capture time is estimated to be about 4.38 to 450.6 μs.

**Figure 4:**
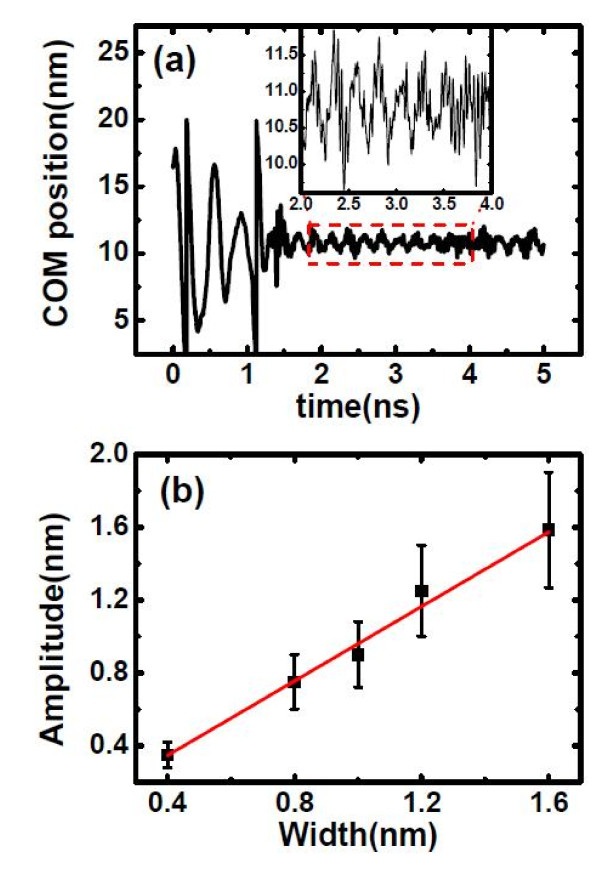
**The positions of COM and the averaged amplitude of oscillation of C_60_**. (**a**) The positions of COM of C_60 _as a function of simulation time under heat flux of 6.5 eV/ps. The inset shows the detailed oscillation of C_60 _in the Z-direction. (**b**) The amplitude of oscillation in the Z-direction as a function of the width of cold thermostat.

In our simulations, the oscillating motions in the Z-direction were observed when the C_60 _cluster was trapped by the thermal gradient, as shown in Figure 4a. In order to investigate the relationship of the amplitude of oscillation and the width of cold thermostat, we conduct a series of simulations with various cold thermostat widths ranging from 0.4 to 1.6 nm. The calculated amplitudes are obtained by averaging the amplitudes of oscillation curves when the C_60 _cluster was trapped by the thermal gradient (see inset in Figure 4a). We can see that the magnitude of oscillation amplitude of the C_60 _cluster is almost the same as the width of the cold region, and an approximate linear relationship between the amplitude (*A*, in Ångström) and the width of the cold thermostat (*W*_c_, in Ångström) was obtained:

(10) $$A=k_{A}W_{\text{c}},$$

where *k_A _*is 1.02. Therefore, a narrower thermostat is preferable if we want to accurately control the nanoparticles by thermal gradient.

**Figure 5:**
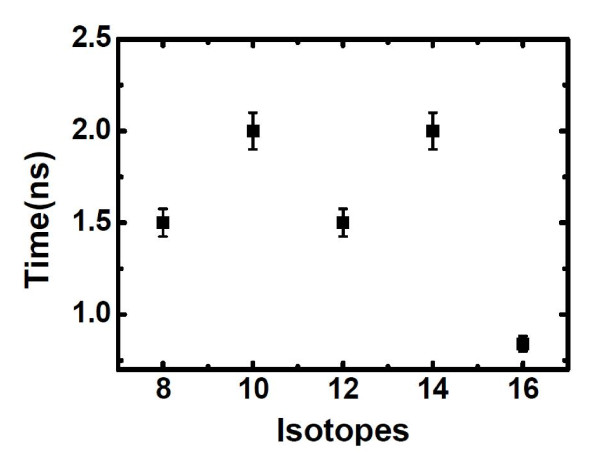
**Trapping time of C_60 _as a function of carbon isotopes (in atomic mass)**. The unit of time is nanoseconds, and the unit of carbon isotopes is atomic mass.

**Figure 6:**
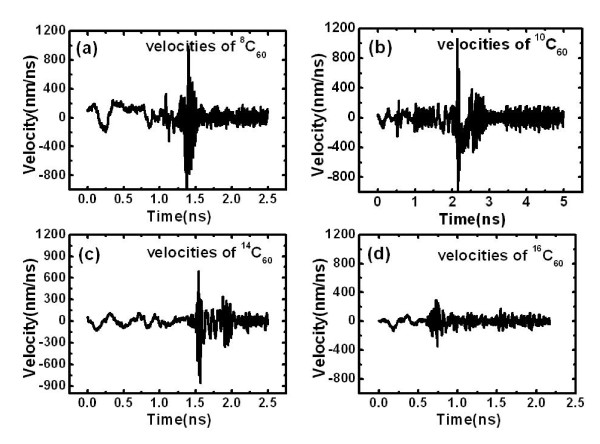
**Velocities of C_60 _consist of different carbon isotopes as a function of time**. (**a**) ^8^C_60_, (**b**) ^10^C_60_, (**c**) ^14^C_60_, and (**d**) ^16^C_60_.

The restraining of encapsulated molecular clusters with different masses by the thermal gradient is also considered by replacing carbon atoms in the C_60 _cluster with various kinds of carbon isotopes. Here, the C_60 _cluster is employed as a typical case of molecular cluster enclosed into CNT, and the C_60 _clusters with different masses are applicable for the similar cases of molecules with various masses. According to the equation of the escaping velocity (Equation 7), both mass and velocity of the molecule play an important role in the molecule's escape from the thermal gradient potential valley. In order to further elucidate the relationship of the trapping time and the mass of molecular cluster, C_60 _clusters consisted of different carbon isotopes, namely ^8^C, ^10^C, ^14^C, and ^16^C, are considered, and the results are shown in Figure 5. Generally, the C_60 _cluster can be trapped by thermal gradient induced by a heat flux of 6.5 eV/ps, and the trapping time is in the region of 1.5 to 2.0 ns for the cases of ^8^C, ^10^C, ^12^C, and ^14^C while less than 1.0 ns for the case of ^16^C. We then plotted their velocity profiles from the simulations in Figure 6 to examine whether they are coupled with the mass of C_60_. We can see that the larger the mass of the C_60 _cluster, the smaller the velocity. For example, compared with the C_60 _clusters consisted of ^8^C, ^10^C, ^12^C, or ^14^C, the thermophoretic force induces a much smaller velocity for the ^16^C_60 _cluster. Its maximum velocity is about 300 nm/ns, and its velocities after being trapped are reduced to about 100 nm/ns. This explains why it is easier to trap. We can also see that in the same conditions, it does not necessarily take longer for the heavier clusters to be trapped than the lighter ones if their initial velocities are induced by thermal fluctuation [33]. Nevertheless, for the clusters with the same velocities, it will be harder to trap the heavier clusters according to our escaping relationship.

## Conclusions

In summary, we propose a new approach to manipulate nanoparticles by imposing a thermal gradient on the system. We extend the study of thermophoresis in nanotechnology from the continuous linear motion of nanoparticles that has been actively studied to the position restraining of nanoparticles by designing a one-dimensional thermal potential barrier. Nanoparticles will be pushed to the cold region by thermophoretic force and be restrained in the cold region with a small vibration of ± 1.0 nm along the tube's axial direction. Moreover, we further extend the idea of nanoparticle restraining to nanomanipulation. When the nanoparticle is restrained by thermal gradient potential valley, nanomanipulation can be achieved by translating thermal sources, and the nanoparticle will move following the same trace of the cold source. A nanoparticle inside CNT can therefore be manipulated on demand by thermophoretic force without a contact with it. The study of thermophoresis by nanorestrain and nanomanipulation will lead to a much wider usage of thermophoresis in the nanosystem and reveal the great potential applications of thermophoresis in nanodesign, mass transport, drug delivery, etc. Experimental work is thus called for to realize the thermophoretic utilization of nanomanipulation and nanodesign in potential applications.

## Competing interests

The authors declare that they have no competing interests.

## Authors' contributions

NW carried out the molecular dynamics simulation and drafted the manuscript. H-QW participated in the organization of the project and discussion of the results, and revised the manuscript. J-CZ organized the project, analyzed the results, and revised the manuscript. All authors read and approved the final manuscript.

## Supplementary Material

Additional file 1 **Supporting information**. In this supplemental information, we describe the method we used to evaluate the energy of one-dimensional potential valley, which is induced by the thermal gradient. Click here for file
