# Supplementary material for: Nanoparticle manipulation by thermal gradient
Source: Nanoscale Res Lett. 2012 Feb 26;7(1):154. doi: 10.1186/1556-276X-7-154 (PMC3306267; doi:10.1186/1556-276X-7-154)
Supplement: Additional file 1 — Supporting information. In this supplemental information, we describe the method we used to evaluate the energy of one-dimensional potential valley, which is induced by the thermal gradient. [file 1556-276X-7-154-S1.DOC]

**Supporting Information**

# Nanoparticle Manipulation by Thermal Gradient

### Ning Wei^1^, Hui-Qiong Wang^1,2*^, Jin-Cheng Zheng^1,3*^

^1^ Department of Physics, and Institute of Theoretical Physics and Astrophysics, Xiamen University, Xiamen 361005, China.

^2^ Fujian Key Lab of Semiconductor Materials and Applications, Xiamen University, Xiamen 361005, People’s Republic of China

^3^ Fujian Provincial Key Laboratory of Theoretical and Computational Chemistry, Xiamen University, Xiamen 361005, China.

* Corresponding author

In this supplemental information, we describe the method we used to evaluate the energy of one-dimensional potential valley, which is induced by the thermal gradient. The energy of one-dimensional potential valley is defined as: the needed energy of taking C_60_ from the bottom of potential valley (the cold region) to the top of the potential valley (the hot region). It is calculated by the path integral of the thermophoretic force on C_60_ from the potential valley to the top:

$E_{t}=\int F_{t}dz$, (1)

where $F_{t}$ is the thermophoretic force.

According to the approximate linear relationship of $F_{t}$ and $\frac{dT}{dz}$from our main text, thermophortic force is expressed as:

$F_{t}=k\times\frac{dT}{dz}$, (2)

so the energy of the potential can be written as:

$E_{t}=k\int\frac{dT}{dz}dz$. (3)

The temperature profiles are obtained within 0.6 ns upon the formation of thermal gradient. This is because, as the simulation time increases, the position of C_60_ will be confined by the thermal potential valley, which will enhance the interactions between C_60_ and CNT and thus affect the temperature distribution. In Fig. S1, we present the temperature profiles and their corresponding thermal gradients under various heat fluxes. It can be seen that, the greater the heat flux, the larger thermal gradient it induces.

The thermal gradient distributions on a carbon nanotube are symmetry along the axial direction, so they are polynomial fitted only from the middle to the left on carbon nanotube, as shown in Fig.S2.


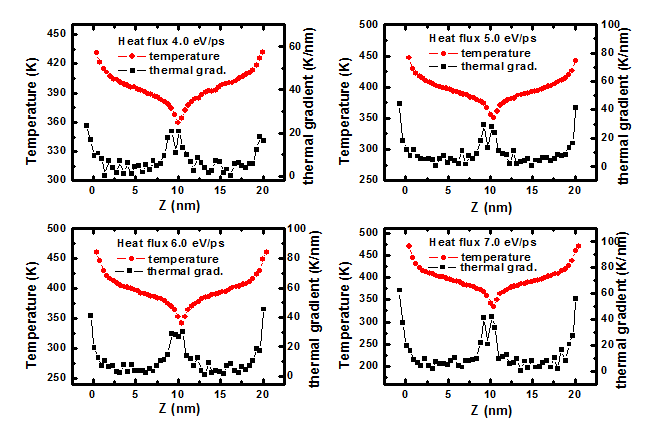


**Figure S1.** Temperature profile and thermal gradient of carbon nanotube subjected to different heat fluxes.


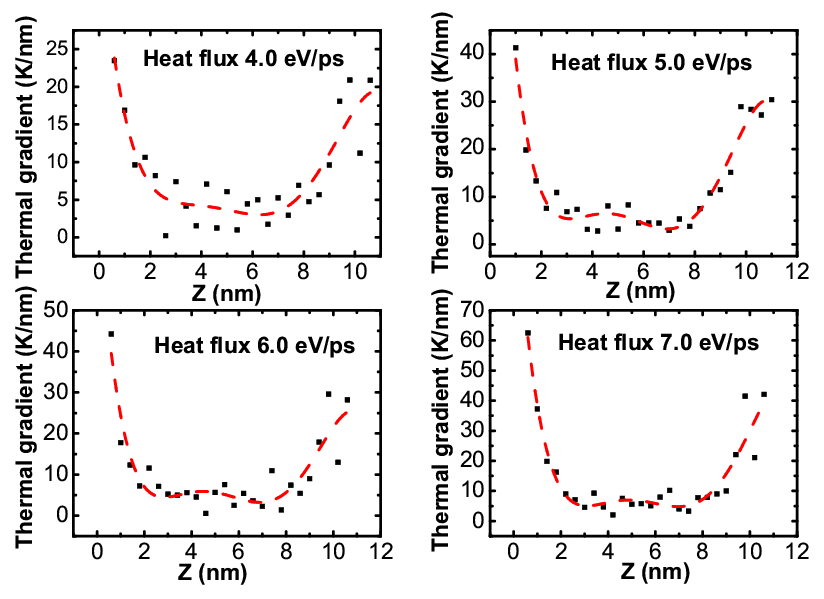


**Figure S2.** The thermal gradient profiles on nanotubes (from middle to the left end) under different imposed heat fluxes are fitted by polynomial fitting formula (dash line) ($\frac{dT}{dz}$=a_0_+a_1_z+a_2_z^2^+a_3_z^3^+a_4_z^4^+a_5_z^5^), where for heat flux=4.0 eV/ps, a_0_=41.51242, a_1_=-37.30735, a_2_=14.66012, a_3=_-2.7736, a_4_=0.24678, a_5_=-0.00812; for heat flux=5.0 eV/ps, a_0_=76.11139, a_1_=-77.92226, a_2_=31.60556, a_3=_-5.91538, a_4_=0.51105, a_5_=-0.01631; for heat flux=6.0 eV/ps, a_0_=116.29771, a_1_=-114.40056, a_2_=44.51172, a_3=_-8.09663, a_4_=0.68598, a_5_=-0.02161; for heat flux=7.0 eV/ps, a_0_=113.31779, a_1_=-109.6412, a_2_=41.56869, a_3=_-7.31067, a_4_=0.59604, a_5_=-0.01793.
